# Trace element partitioning between pyrochlore, microlite, fersmite and silicate melts

**DOI:** 10.1186/s12932-020-00072-w

**Published:** 2020-08-24

**Authors:** Stephan Klemme, Jasper Berndt

**Affiliations:** Institut für Mineralogie, Universität Münster, Corrensstraße 24, 48149 Münster, Germany

**Keywords:** Pyrochlore, Microlite, Nb, Ta, Ore deposit, Fersmite, Trace element, Partition coefficients, Alkaline rocks, Experimental petrology, LA-ICP-MS, Electron microprobe

## Abstract

We present experimentally determined trace element partition coefficients (D) between pyrochlore-group minerals (Ca_2_(Nb,Ta)_2_O_6_(O,F)), Ca fersmite (CaNb_2_O_6_), and silicate melts. Our data indicate that pyrochlores and fersmite are able to strongly fractionate trace elements during the evolution of SiO_2_-undersaturated magmas. Pyrochlore efficiently fractionates Zr and Hf from Nb and Ta, with D_Zr_ and D_Hf_ below or equal to unity, and D_Nb_ and D_Ta_ significantly above unity. We find that D_Ta_ pyrochlore-group mineral/silicate melt is always higher than D_Nb_, which agrees with the HFSE partitioning of all other Ti–rich minerals such as perovskite, rutile, ilmenite or Fe-Ti spinel. Our experimental partition coefficients also show that, under oxidizing conditions, D_Th_ is higher than corresponding D_U_ and this implies that pyrochlore-group minerals may fractionate U and Th in silicate magmas. The rare earth element (REE) partition coefficients are around unity, only the light REE are compatible in pyrochlore-group minerals, which explains the high rare earth element concentrations in naturally occurring magmatic pyrochlores.

## Introduction

To understand the behavior of trace elements in igneous rocks, trace element partition coefficients between minerals and melts are needed. Over the last decades, numerous experimental studies focused on the trace element partitioning between major rock forming minerals and melts [1], but few experimental partition coefficients are available for accessory phases such as rutile, ilmenite, spinel or apatite in basaltic compositions [2–12] and even less data are available for accessory mineral phases such as perovskite or pyrochlore in alkaline rock compositions [13]. As the aforementioned accessory mineral phases commonly occur in alkaline igneous rocks, they may exert a strong control on the trace element evolution of alkaline magmas.

In this study we focus on the Nb- and Ta-rich accessory phases pyrochlore, microlite, and fersmite, which are accessory phases in alkaline silicate rocks [14–20] and, perhaps more commonly, carbonatites [18, 21–23]. Note that these mineral phases are the most important hosts for Nb and Ta in ore deposits (e.g., [18, 24–27].

The pyrochlore-group of minerals in silicate rocks and carbonatites encompasses a very large und very complex group of minerals [28]. The general formula of minerals of the pyrochlore supergroup is A_2_B_2_O_7_, where the A-site is often occupied by monovalent or divalent cations, and the B-site is mainly occupied by pentavalent cations such as Nb or Ta. Pyrochlore *senso stricto* is a mineral in which the B-site is occupied by Nb, and microlites are minerals in which the B-site is occupied by Ta [28]. We do not aim to describe the entire compositional variability of pyrochlores and related minerals in alkaline rocks, and we would like to refer the interested reader to excellent papers by Mitchell, Chakhmouradian, and others [18, 21, 22, 26, 29–33]. However, common accessory mineral phases in carbonatites and alkaline silicate rocks are Ca-Na pyrochlores that can (and often do) incorporate almost all geochemical indicator elements, including the rare earth elements (REE), the large ion lithophile elements (e.g., K and Ba), and, as they are Nb- and Ta-rich minerals, also the high field strength elements Nb, Ta, Zr, and Hf (e.g., [21, 22, 26, 30, 31, 33, 34].

As there are, to our knowledge, no previous experimental studies on the partitioning of trace elements between pyrochlore-group minerals and melts, we set out to determine trace element partition coefficients between pyrochlore (Ca_2_Nb_2_O_7_), microlite (Ca_2_Ta_2_O_7_), fersmite (CaNb_2_O_6_), and silicate melts. The long-term objective of this work is to determine the effects of temperature, pressure, and perhaps most importantly, chemical composition of minerals on trace element partition coefficients. Hence future experiments will be extended towards systems with carbonate melts. Our experiments presented here were done in simplified chemical compositions, and must hence be considered as a first step towards a better understanding of trace element partitioning in complex natural systems where pyrochlore-group minerals occur.

## Experimental and analytical methods

### Starting materials

To ensure the nucleation and growth of pyrochlore-group minerals and fersmite to a reasonable size and, thus, enabling in-situ analysis by laser ablation inductively-coupled plasma mass spectrometry (LA-ICP-MS), we conducted a few exploratory experiments to establish chemical compositions which precipitated pyrochlore-group minerals and fersmite. In order to avoid possible experimental problems related to Fe-loss, all starting materials did not contain Fe. Starting material SM_Pyro1 is a composition in the system CaO-Na_2_O-Al_2_O_3_-SiO_2_-Nb_2_O_5_-F, and HWM in the system CaO-Na_2_O-Al_2_O_3_-SiO_2_-Ta_2_O_5_. The choice of this rather simple chemical composition was made after our reconnaissance experiments in more complex systems yielded only small Nb-mineral crystals (< 15 µm), which were impossible to analyze with our LA-ICP-MS set-up. Both starting materials were prepared from analytical grade oxides, hydroxides, and carbonates (Table 1), which were ground in an agate mortar under ethanol. The starting material mixtures were heated in air at 1000 °C for 3 h in order to ensure complete decarbonation. Subsequently, the mixtures were heated to temperatures well above 1600 °C (10 min), i.e. above the liquidus in these systems, quenched, and the resulting glasses were re-ground to fine powders. All starting materials (except in run SE_Pyro2, see below) were doped with a trace element mixture (SKM-TE1) containing the following elements: La, Ce, Nd, Sm, Gd, Dy, Yb, Lu, Y, Li, Sr, Ba, Rb, Sc, Mn, Ni, Co, Mo, Te, Ga, Ge, In, Cr, Zr, Nb, Hf, Ta, Pb. U and Th were added separately using 1000 µg/g ICP-AES standard (Alfa Aesar, in diluted nitric acid) solutions. The starting material compositions are given in Table 1. Note that run SE_Pyro2 was an exploratory run to which no trace elements were added. The doped starting materials were subsequently dried and denitrified in a laboratory-type drying cabinet at 110 °C (12 h).

**Table 1:** Starting materials

	SM_Pyro1	HWM	SKM-TE1							
	wt.%	wt.%		wt.%		wt.%		wt.%		wt.%
Na_2_O	1.1	0.9	La	2.8	Lu	3.0	Sr	2.2	Ga	1.6
CaO	24.8	28.9	Ce	2.6	Y	2.3	Pb	6.8	Ge	7.6
Al_2_O_3_	6.7	3.8	Nd	3.6	Zr	6.0	Mn	2.3	In	2.6
SiO_2_	19.7	21.6	Sm	7.0	Hf	6.6	Ni	3.0	Cr	1.6
Nb_2_O_5_	44.7	–	Gd	2.8	Nb	3.0	Co	5.7	Li	0.4
Ta_2_O_5_	–	44.8	Dy	2.7	Ta	4.6	Mo	5.7	Ba	2.9
F	3.0	–	Yb	2.3	Rb	4.2	Te	1.5	Sc	2.4
total	100	100								

Note that the trace element mixture SKM-TE1 was added to both starting materials SM_Pyro1 and HWM, only run SE_Pyro2 was run without trace elements. See text for details

### Experimental techniques

All experiments were run in conventional 1-atmosphere vertical furnaces in air [11], using the Pt-wire loop technique (e.g., [11, 13, 35–37]. To prepare the loops, we mixed about 20 mg of starting material powder into a viscous slurry with a synthetic glue, and we loaded this mixture onto a 0.1 mm diameter Pt-wire loop. The samples were then introduced into the hotspot of a vertical alumina tube furnace (Gero GmbH, Germany). Temperature was controlled with a thermocouple external to the alumina tube by a Eurotherm controller, limiting fluctuations to within 1 °C. The temperature was additionally monitored with a Type B thermocouple (Pt_70_Rh_30_-Pt_94_Rh_6_) close to the sample. The runtime (at final run temperature) varied between 20 and 120 h (see Table 2 for details). After quenching of the experimental charges, the samples were embedded in epoxy resin and polished using several different diamond pastes, and carbon coated for qualitative and quantitative analysis.

**Table 2:** Experimental conditions and run products

Run no	T/°C start	Cooling rate °/min	T/°C end	Duration (h) at T end	Starting material	Phases present
SE_Pyro2	1400	100	1300	24	SM_Pyro1	Fer, Nb-pyr, melt
SE_Pyro3	1400	20	1300	24	SM_Pyro1	Fer, Nb-pyr, melt
SE_Pyro5	1400	10	1260	24	SM_Pyro1	Fer, Nb-pyr, melt
SE_Pyro6	1400	5	1240	120	SM_Pyro1	Fer, Nb-pyr, melt
HW1	1400	0.1	1300	28	HWM	Ta-pyr, melt
HW2	1350	0.2	1200	20	HWM	Ta-pyr, melt
HW3	1400	–	1400	46	HWM	Ta-pyr, melt

Fer=Ca-Nb-fersmite; Nb-pyr=Ca-Nb-pyrochlore; melt=quenched melt; Ta-pyr=Ca-Ta-microlite

Note that in run HW3 no cooling rate was used. Note also that fersmite in runs SE_Pyro3, and pyrochlores in SE_Pyro2 were acicular and too small for EMPA analysis. All experiments were run in air and at atmospheric pressure. T/°C start: T at which the samples were inserted into the furnace, T/°C end: The temperature of the run after the cooling rate; cooling rate: The samples were cooled from T_start_ to T_end_, and the runs were held at T_end_ for at least 20 h (duration (h) at T_end_

### Analytical techniques

The experimental run products were examined with a JEOL6610LV scanning electron microscope with an EDX system and the major element concentrations of all phases were determined with a 5-spectrometer JEOL JXA 8530F electron microprobe analyzer (EMPA) at the Institut für Mineralogie at the University of Münster. Mineral phases were analyzed using beam spot sizes between 2–5 µm at a current of 15 nA. Counting times were 20 s on the peak and 10 s on the background. Glasses and quenched melts were analyzed with a 10 µm defocused beam and counting times were 5 s on the peak and 3 s on the background to minimize loss of volatile elements. Matrix-matched minerals were used as reference materials. Additionally, a set of secondary standards was measured together with the unknowns to monitor external precision and accuracy [38]. Trace elements were analyzed using a LA-ICP-MS system at the University of Münster that consisted of an Element 2 (ThermoFisher Scientific) SF-ICP-MS connected to a 193 nm ArF excimer laser ablation system (Teledyne Photon Machines Analyte G2). Laser repetition rate was 5 Hz using an energy density of about 4 J/cm^2^. Prior to sample analyses, the system was tuned with the NIST SRM 612 standard for high sensitivity, stability, and low oxide rates (^232^Th^16^O/^232^Th < 0.1%). Ablation time was 40 s and the background was measured for 20 s prior to sample ablation. Spot size was 12–25 µm for pyrochlores and fersmite crystals and melts, and 35 µm for the reference materials. ^43^Ca was used for internal calibration and NIST SRM 612 was chosen as the external standard using concentration values given in the GeoRem database, version 26/2019 [39]. Groups of 8–17 spot analyses were bracketed by three standard analyses to monitor instrumental drift. All concentrations were calculated using the Glitter software (version 4.4.4 [40]. Standard reference glasses BCR2-G, BIR1-G, and BHVO2-G were analyzed as monitor for precision and accuracy for silicate phases during the course of this study. Obtained results match the published range of concentrations given in the GeoReM database [39].

## Results and discussion

### Experimental results

**Fig. 1:**
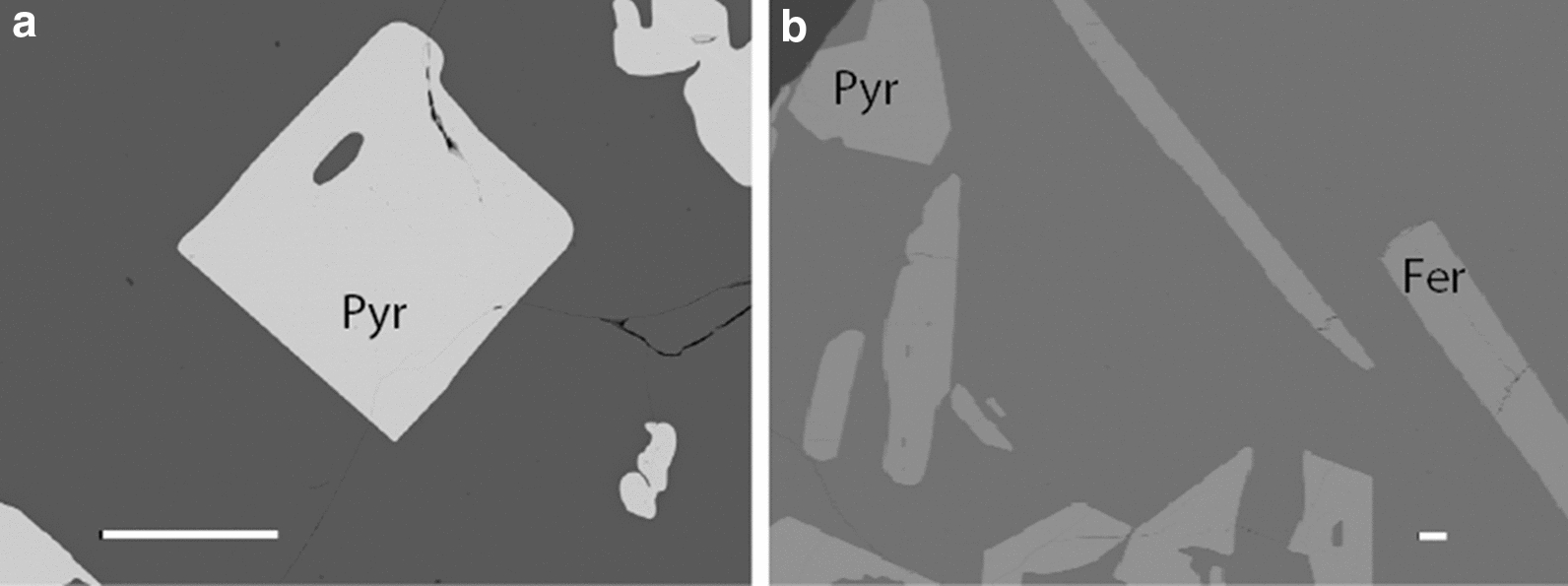
Back-scattered electron images taken with the SEM. The white horizontal scale bar is 100 µm in both images. A: run product HW2 contains pyrochlore surrounded by quenched melt. B: run SE_Pyro_6 contains both fersmite (fer), pyrochlore (pyr), and quenched melt

The experiments (Table 2) yielded euhedral-to-subhedral pyrochlore and fersmite crystals from < 15 µm up to about 150 µm across. In some runs, pyrochlore or fersmite crystal were thin and acicular so that they could not be analyzed with LA-ICPMS (c.f., Table 2) No other crystals were observed. The melt quenched to a dark and inclusion-free homogeneous glass. Electron microprobe and LA-ICP-MS analyses of pyrochlore and fersmite crystals and quenched melts indicate major- and trace element homogeneity, which is taken as evidence for the attainment of equilibrium between crystals and melts in our runs. Representative run products are shown in Fig. 1.

We would like to note that the exploratory experiment SE_Pyro2 was run with a starting material that did not contain added trace elements. Consequently, the trace element concentrations in both minerals and quenched melts in this run are substantially lower than in all other experiments (Table 2). However, the partition coefficients of this run are in excellent agreement with D’s from the other runs (see discussion below, Table 4), which confirms the quality of our trace element analyses at very low concentrations, and also that Henry’s law has been attained [41].

### Analytical results

Major, minor and trace elements of all phases were analyzed using EPMA and LA-ICPMS techniques, and the analytical results are given in Tables 3 and 4. Note that some of the crystals (Table 2) were too small to be analyzed.

**Table 3:** Maj or element compositions of minerals and melts

n	5		8		10		10					
	SE_Pyro2		SE_Pyro2		SE_Pyro3		SE_Pyro3					
	Fer		Melt		Nb-Pyr		Melt					
	av	Stdev	av	Stdev	av	Stdev	av	Stdev				
Na_2_O	n.d		0.4	0.04			0.24	0.08				
Al_2_O_3_	n.d		5.4	0.5			5.2	0.1				
CaO	17.4	0.5	31.3	0.8	28.6	0.4	31.3	0.8				
SiO_2_	n.d		28.2	0.2			28.2	0.2				
Ta_2_O_5_	n.a		n.a		n.a		n.a					
Nb_2_O_5_	80.8	0.03	30.7	0.1	68.4	0.2	30.7	0.1				
F	0.9	0.2	3.5	0.5	2.6	0.4	2.6	0.2				
Total	99.1		99.50		99.60		98.2					

n	3		10		2		8		10			
	SE_Pyro5		SE_Pyro5		SE_Pyro6		SE_Pyro6		SE_Pyro6			
	Fer		Melt		Fer		Nb-Pyr		Melt			
	av	Stdev	av	Stdev	av	Stdev	av	Stdev	av	Stdev		
Na_2_O	n.d		n.d		n.d		n.d		n.d			
Al_2_O_3_	n.d		6.1	0.9	n.d		n.d		6.3	0.1		
CaO	17.1	0.3	32.5	0.5	17.3	0.5	29.0		32.7	0.2		
SiO_2_	n.d		32.3	0.4	n.d		n.d		33.7	0.3		
Ta_2_O_5_	n.a		n.a		n.a		n.a		n.a			
Nb_2_O_5_	82.8	0.4	23.1	0.7	80.8	0.5	67.7		21.8	0.4		
F	0.5	0.2	4.2	0.6	0.8	0.3	2.4		3.1	0.3		
Total	100.4		98.2		98.9		99.2		97.6			

n	10		10		10		10		10		10	
	HW1				HW2				HW3			
	Ta-pyr		Melt		Ta-pyr		Melt		Ta-pyr		Melt	
	av	Stdev	av	Stdev	av	Stdev	av	Stdev	av	Stdev	av	Stdev
Na_2_O	0.06	0.03	0.4	0.1	0.05	0.03	0.52	0.03	0.12	0.03	4.2	0.1
Al_2_O_3_	0.04	0.01	6.5	0.1	0.04	0.02	5.78	0.11	0.03	0.02	14.7	0.1
CaO	20.1	0.2	37.2	0.5	20.1	0.1	35.2	0.3	19.9	0.2	25.0	0.4
SiO_2_	0.04	0.03	41.1	0.7	0.05	0.04	37.2	0.2	0.04	0.03	45.0	0.4
Ta_2_O_5_	79.3	0.5	15.5	1.0	79.2	0.6	21.7	0.2	79.9	0.8	10.1	0.8
Nb_2_O_5_	0.13	0.05	0.09	0.04	0.13	0.04	0.1	0.1	0.10	0.05	0.02	0.01
Total	99.6	0.5	100.7	2.4	99.6	0.9	100.4	0.9	100.0	1.1	99.0	1.8

Major element composition of minerals and quenched melts (Fer = Ca-Nb-fersmite, Nb-pyr = Ca-Nb-pyrochlore, melt = quenched melt, Ta—pyr = Ca-Ta-microlite) analyzed with EMPA. *n* number of analyses, *n.d.* not detected, *n.a.* not analyzed. Note that fersmite occurred in all runs with the SE_Pyro1 starting materials (Table 1) but in most runs these crystals were too small too be analyzed, and we only present data from runs SE_Pyro5 and SE_Pyro6.

**Table 4:** Trace element compositions of minerals and melts, and calculated partition coefficients

	HW1									
n	7		6							
	Ta-Pyr	Stdev	Melt	Stdev	D Ta-pyr HW1	Stdev				
Li	1.0	0.1	12.7	0.3	0.08	0.01				
Sc	205	22	1100	14	0.19	0.02				
Mn	101	11	290	1	0.35	0.04				
Co	205	37	1290	10	0.16	0.03				
Ni	76	16	641	4	0.12	0.03				
Ga	21	11	791	13	0.03	0.01				
Ge	77	10	250	4	0.31	0.04				
Sr	187	3	477	3	0.39	0.01				
Y	708	105	707	4	1.0	0.1				
Zr	896	130	906	8	1.0	0.1				
Nb	999	92	777	12	1.3	0.1				
Mo	0.20	0.18	12.8	0.2	0.02	0.01				
Ba	33	9	736	7	0.04	0.01				
La	1111	173	448	6	2.5	0.4				
Ce	1070	361	191	3	5.6	1.9				
Nd	1695	389	490	8	3.5	0.8				
Sm	1408	323	466	4	3.0	0.7				
Gd	675	127	317	3	2.1	0.4				
Dy	565	91	372	2	1.5	0.2				
Yb	324	37	487	3	0.7	0.1				
Lu	558	64	1048	11	0.5	0.1				
Hf	1390	299	782	14	1.8	0.4				
Ta	590,712	27,309	121,645	5874	4.9	0.3				
Th	0.5	0.2	0.06	0.01	7.8	3.1				
U	1.6	0.3	4.0	0.1	0.4	0.1				

	HW2									
n	8		6							
	Ta-Pyr	Stdev	Melt	Stdev	D Ta-pyr HW2	Stdev				
Li	1.4	0.2	14.3	0.4	0.10	0.02				
Sc	187	25	985	10	0.19	0.03				
Mn	105	4	282	7	0.37	0.02				
Co	208	18	1197	12	0.17	0.01				
Ni	77	11	581	6	0.13	0.02				
Ga	18	5	705	9	0.03	0.01				
Ge	70	8	60	2	1.18	0.13				
Sr	180	3	448	5	0.40	0.01				
Y	678	73	674	16	1.01	0.11				
Zr	834	116	933	16	0.89	0.13				
Nb	899	45	882	10	1.02	0.05				
Mo	0.09	0.04	6.2	0.2	0.015	0.007				
Ba	30	4	660	7	0.05	0.01				
La	1067	74	481	5	2.2	0.2				
Ce	1053	203	240	4	4.4	0.9				
Nd	1693	212	543	7	3.1	0.4				
Sm	1388	178	503	8	2.8	0.4				
Gd	662	74	329	5	2.0	0.2				
Dy	550	57	371	6	1.5	0.2				
Yb	318	30	450	7	0.7	0.1				
Lu	545	45	964	15	0.57	0.05				
Hf	1326	202	848	18	1.6	0.2				
Ta	603,553	6796	167,305	2991	3.6	0.1				
Th	0.5	0.1	0.080	0.004	5.9	1.5				
U	0.9	0.1	3.56	0.03	0.26	0.02				

	HW3									
n	6		5							
	Ta-Pyr	Stdev	Melt	Stdev	D Ta-pyr HW3	Stdev				
Li	3.2	0.6	68	21	0.05	0.02				
Sc	112.8	48.9	1053	333	0.11	0.06				
Mn	66.5	19.5	517	152	0.13	0.05				
Co	109.5	43.9	3013	988	0.04	0.02				
Ni	33.4	18.1	1409	456	0.024	0.015				
Ga	10.5	1.2	1915	608	0.005	0.002				
Ge	85.3	8.3	1340	419	0.06	0.02				
Sr	196.5	6.5	904	291	0.22	0.07				
Y	717.7	41.7	235	69	3.1	0.9				
Zr	665.9	127.1	208	59	3.2	1.1				
Nb	831.0	13.7	487	148	1.7	0.5				
Mo	1.2	0.1	70	23	0.017	0.006				
Ba	34.1	2.2	1843	570	0.018	0.006				
La	1417	231	509	154	2.8	1.0				
Ce	1527	269	87	24	17.6	5.8				
Nd	2235	305	332	93	6.7	2.1				
Sm	1770	171	251	71	7.1	2.1				
Gd	778	35	138	39	5.6	1.6				
Dy	608	18	138	40	4.4	1.3				
Yb	304	31	148	43	2.0	0.6				
Lu	493	66	319	94	1.5	0.5				
Hf	1054	300	367	104	2.9	1.2				
Ta	589,199	9317	87,900	24,870	6.7	1.9				
Th	0.8	0.2	0.10	0.03	7.8	3.0				
U	1.5	0.3	12	4	0.12	0.04				

	SE_Pyro3									
n	5		5							
	Nb-pyr	Stdev	Melt	stdev	D Nb-pyr/melt SE3	Stdev				
Li	3.5	0.3	13	1	0.27	0.03				
Sc	315	151	1815	45	0.17	0.08				
Mn	102	34	482	4	0.21	0.07				
Co	298	157	1852	96	0.16	0.09				
Ni	158	90	977	47	0.16	0.09				
Ga	196	104	1125	64	0.17	0.09				
Ge	40	3	50	28	0.8	0.5				
Sr	547	10	618	12	0.88	0.02				
Y	893	53	1400	60	0.64	0.05				
Zr	697	139	2050	74	0.34	0.07				
Nb	489,667	38,502	273,134	6537	1.8	0.1				
Mo	10	1	27	5	0.37	0.09				
Ba	265	68	944	18	0.28	0.07				
La	1895	259	911	74	2	0.3				
Ce	994	70	670	34	1.5	0.1				
Nd	2376	314	1223	82	1.9	0.3				
Sm	1791	169	1098	56	1.6	0.2				
Gd	843	55	678	34	1.2	0.1				
Dy	665	18	737	34	0.9	0.05				
Yb	396	51	820	30	0.48	0.06				
Lu	762	133	1847	81	0.41	0.07				
Hf	901	105	1980	116	0.45	0.06				
Ta	4361	947	1142	100	3.8	0.9				
Th	167	6	196	11	0.85	0.06				
U	23	12	108	31	0.2	0.1				

	SE_Pyro4									
n	5		5							
	Nb-pyr	Stdev	melt	Stdev	D Nb-pyr/melt SE4	Stdev				
Li	5.3	0.4	11.7	0.5	0.97	0.09				
Sc	551	182	1822	18	0.82	0.27				
Mn	127	59	514	3	0.7	0.3				
Co	363	270	2034	52	0.58	0.43				
Ni	188	125	1032	25	0.6	0.4				
Ga	196	147	1008	23	0.5	0.4				
Ge	123	67	288	191	2.3	2.0				
Sr	568	18	606	18	1.31	0.06				
Y	825	168	1421	24	1.10	0.20				
Zr	1180	190	2058	47	1.20	0.20				
Nb	481,096	60,016	268,189	9708	1.50	0.20				
Mo	8.5	0.8	18	4	0.8	0.2				
Ba	339	108	962	20	0.9	0.3				
La	1916	147	841	21	1.6	0.1				
Ce	932	59	643	10	1.43	0.09				
Nd	2359	141	1176	28	1.6	0.1				
Sm	1681	94	1065	21	1.47	0.09				
Gd	784	73	668	13	1.40	0.10				
Dy	609	91	739	15	1.20	0.20				
Yb	379	118	835	14	0.90	0.30				
Lu	753	279	1919	64	0.90	0.30				
Hf	1561	247	1997	70	1.4	0.2				
Ta	3862	1055	1084	85	1.6	0.5				
Th	199	22	189	4	1.41	0.2				
U	38	25	133	44	0.91	0.7				

	SE_Pyro5									
n	5		5							
	Fer	Stdev	melt	Stdev	D fer/melt SE5	Stdev				
Li	4.2	1.0	13.6	0.5	0.31	0.07				
Sc	388	214	2292	14	0.17	0.09				
Mn	94	57	602	5	0.16	0.09				
Co	328	253	2281	24	0.1	0.08				
Ni	160	84	1144	14	0.14	0.07				
Ga	187	145	1194	12	0.2	0.1				
Ge	13	2	51	9	0.24	0.06				
Sr	175	76	736	16	0.20	0.10				
Y	918	166	1609	40	0.60	0.10				
Zr	1269	248	2462	81	0.50	0.10				
Nb	474,314	123,606	253,661	11,699	1.9	0.5				
Mo	13	3	20	0	0.6	0.1				
Ba	178	138	1187	39	0.1	0.08				
La	483	74	973	35	0.5	0.08				
Ce	349	44	719	19	0.49	0.06				
Nd	1034	185	1262	34	0.8	0.1				
Sm	973	207	1145	27	0.8	0.2				
Gd	611	137	725	19	0.8	0.2				
Dy	582	133	818	28	0.7	0.2				
Yb	454	75	950	31	0.48	0.08				
Lu	967	137	2200	74	0.44	0.06				
Hf	1334	288	2501	91	0.5	0.1				
Ta	2973	749	812	33	3.7	0.9				
Th	95	20	227	7	0.42	0.09				
U	9	3	163	12	0.05	0.02				

	SE_Pyro6									
n	5		2		5					
	Nb-pyr	Stdev	Fer	Stdev	Melt	Stdev	D Nb-pyr/melt SE6		D fer/melt SE6	
Li	3	–	–	–	10.3	0.2	0.25	–	–	–
Sc	62	9	70	51	2240	11	0.028	0.004	0.03	0.02
Mn	33	9	26	2	570	5	0.06	0.02	0.045	0.0003
Co	43	22	14	3	2191	24	0.02	0.01	0.006	0.001
Ni	13	5	–	–	1115	14	0.012	0.004	–	–
Ga	6.6	1.1	1.2	0.5	1286	19	0.0051	0.0009	0.001	0.0004
Ge	22	2	13	4	23.6	1.0	0.92	0.08	0.5	0.2
Sr	366	7	92	3	781	15	0.47	0.01	0.117	0.005
Y	555	20	1001	314	1539	30	0.36	0.02	0.7	0.2
Zr	319	39	1372	439	2300	45	0.14	0.02	0.6	0.2
Nb	398,834	6729	803,171	23,398	209,001	6675	1.91	0.07	3.8	0.2
Mo	5.7	0.5	11.9	0.6	47.8	1.0	0.12	0.01	0.25	0.01
Ba	98	4	1.7	0.5	1167	29	0.084	0.004	0.0015	0.0005
La	1439	109	343	143	1265	27	1.14	0.09	0.3	0.1
Ce	712	31	282	126	858	21	0.83	0.04	0.3	0.1
Nd	1824	125	1089	361	1504	32	1.21	0.09	0.7	0.2
Sm	1291	75	1119	353	1266	30	1.02	0.06	0.9	0.3
Gd	597	25	742	214	738	16	0.81	0.04	1.0	0.3
Dy	434	12	682	207	796	20	0.55	0.02	0.9	0.3
Yb	208	8	448	170	908	22	0.23	0.01	0.5	0.2
Lu	380	19	896	333	2128	47	0.18	0.01	0.4	0.2
Hf	484	63	1180	579	2343	53	0.21	0.03	0.5	0.2
Ta	4050	593	4490	497	985	18	4.1	0.6	4.6	0.5
Th	108	16	64	54	241	5	0.45	0.07	0.3	0.2
U	2.9	0.8	11	6	81	4	0.04	0.01	0.13	0.08

	SE_Pyro2									
n	2		5							
	Fer	Stdev	melt	Stdev	D fer/melt SE2					
Li	–	–	11.2	0.2	–	–				
Sc	–	–	3.0	0.04	-	–				
Mn	2.5	–	22.7	0.5	0.11	–				
Co	1.3	0.4	32.6	0.4	0.04	–				
Ni	–	–	85.6	3.3	–	–				
Ga	–	–	4.4	0.2	–	–				
Ge	2.6	–	17.6	0.3	0.15	–				
Sr	15.6	1.9	123	1	0.13	–				
Y	5.5	0.44	4.1	0.1	1.3	0.2				
Zr	31	12	80	1	0.38	0.03				
Nb	583,356	17,353	253,850	3380	2.3	0.9				
Mo	10.4	0.9	52.3	0.9	0.2	0.007				
Ba	0.7	0.2	13.5	0.4	0.05	0.01				
La	1.6	0.2	2.55	0.04	0.6	0.1				
Ce	2.5	0.3	3.8	0.1	0.67	0.08				
Nd	3.4	0.4	2.5	0.1	1.3	0.2				
Sm	8.8	0.9	5.5	0.1	1.6	0.2				
Gd	3.1	0.4	1.8	0.1	1.7	0.2				
Dy	2.5	0.3	1.7	0.1	1.5	0.2				
Yb	1.8	0.1	1.9	0.1	1.0	0.1				
Lu	1.7	0.2	1.9	0.1	0.86	0.06				
Hf	1.2	0.6	3.0	0.1	0.41	0.04				
Ta	39.6	0.8	13.5	0.1	2.9	1.3				
Th	0.8	0.1	1.14	0.04	0.7	0.1				
U	0.28	0.04	3.08	0.03	0.09	0.01				

Fer = Ca-Nb-fersmite, Nb-pyr = Ca-Nb-pyrochlore, melt = quenched melt, Ta—pyr = Ca- Ta-microlite, stdev = standard deviation (2 sigma)

#### Partition coefficients

**Fig. 2:**
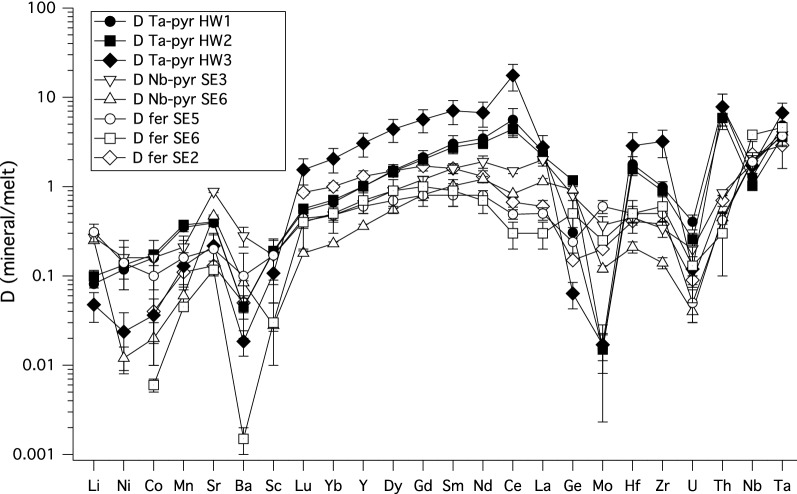
Mineral/melt trace element partition coefficients between pyrochlore-group minerals, (Ca-Ta-microlite (Ta-Pyr) and Ca-Nb pyrochlore (Nb-pyr)), Ca-Nb fersmite (fer), and melts. The elements are arranged in order of increasing valence, i.e. from univalent Li to pentavalent Ta. Minerals and quenched melts were analyzed with LA-ICPMS techniques. See text for details

The trace element concentrations of pyrochlore, microlite, and fersmite crystals and quenched melts were used to calculate trace element partition coefficients (D_i_) using the following expression:

$${\text{D}}_{{\text{i}}} = {\text{ c}}_{{{\text{i}},{\min}}} /{\text{ c}}_{{{\text{i}},{\text{ melt}}}}$$

where D_i_ is the partition coefficient for a trace element *i*, c_i, min_ is the measured concentration of the trace element *i* in the crystal, and c_i, melt_ is the concentration of the trace element *i* in the melt. The calculated trace element partition coefficients together with the propagated uncertainties, are given in Table 4, and are depicted in Fig. 2.

The light element Li is incompatible, with partition coefficients slightly less than unity. The divalent elements Sr and Mn partition evenly between pyrochlore, fersmite and silicate melt, with partition coefficients slightly less than unity, the D’s for Ni and Co are slightly lower (Fig. 2, Table 4). The Ge partition coefficients are all slightly below or close to unity (Fig. 2, Table 4), indicating that Ge follows the geochemical behavior of Si. Sc and Ba partition coefficients cluster around 0.1, with no apparent systematics (Fig. 2).

**Fig. 3:**
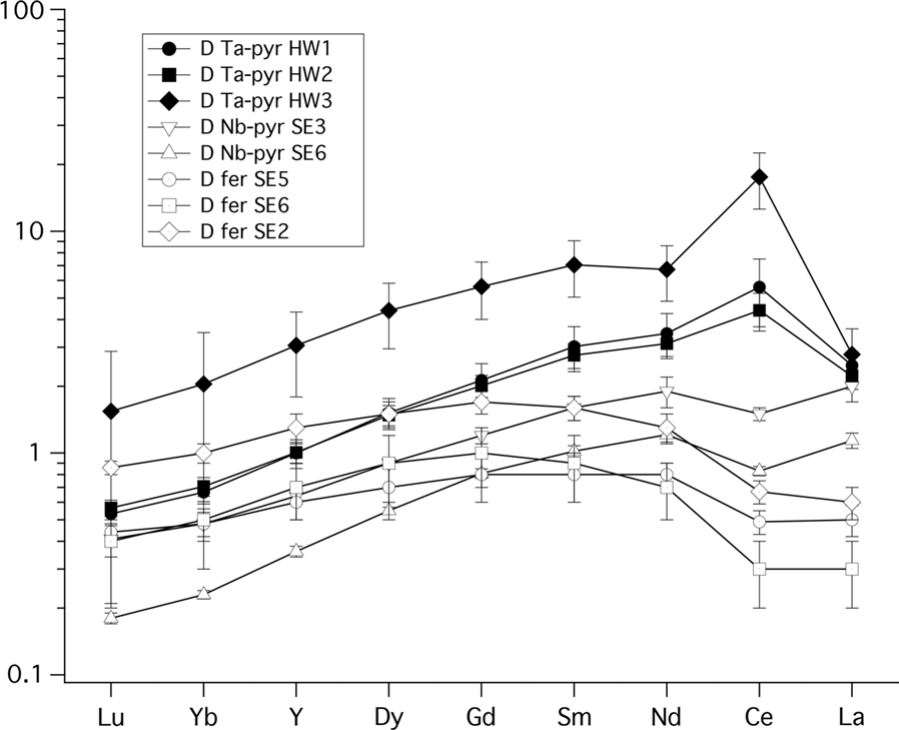
Mineral/melt rare earth element (REE) partition coefficients between pyrochlore-group minerals, (Ca-Ta-microlite (Ta-pyr), and Ca-Nb-pyrochlore (Nb-pyr)), and Ca-Nb-fersmite (fer), and melts. Minerals and quenched melts were analyzed with LA-ICPMS techniques. See text for details

The partition coefficients of the rare earth elements (REE and Y) are depicted in Fig. 3. Our results show that D_REE_ pyrochlore/melt increases with decreasing ionic radius of the REE. The light REE (LREE) La, Ce, Pr, Sm and Gd are compatible in pyrochlore in all runs, regardless of temperature or bulk composition. The D_REE_ pyrochlore/melt of the heavier REE (Yb, Lu, and also Y) are slightly below 1, in run HW3 all D_REE_ are above 1. Our data shows that the D_REE_ of Ta-rich pyrochlores (i.e. the HW1-HW3 runs, black filled symbols in Fig. 3) are systematically higher than the D_REE_ from experiments with Nb-rich pyrochlores (runs SE3 and SE6, open triangles in Fig. 3). As the REE are incorporated on the Ca-site in pyrochlores, the different partitioning may be explained by the different nature of the Ca-site in Ta-rich (i.e. microlite) and Nb-rich pyrochlores, respectively. Crystallographic data show that the size of the Ca-site in Nb-rich pyrochlore is slightly smaller (average Ca-O bond length in pyrochlore of 2.58 Å [42]) than the Ca-site in Ta-pyrochlore (microlite), with an average Ca-O bond length of 2.73 Å [43].

If we only consider the D_REE_ of the Ta-rich microlites (i.e. the HW runs), we find that D_REE_ of HW3, which was run at 1400 °C, is substantially higher than D’s of runs HW2 and HW1 which were run at a final run temperature of 1300° and 1200 °C. However, the D’s of HW1 and HW2 are undistinguishable within the errors and hence the temperature effect on D’s cannot be fully confirmed.

The D_REE_ fersmite/melt show a much flatter and only slightly downwards concave pattern, with D_La_/D_Lu_ very close to unity, and D_REE_ of the middle REE (Nd, Sm, Gd, Dy) slightly higher than the D_LREE_ or D_HREE_, similar to D_REE_ between apatite and melt (e.g., [44]. This clearly indicates that the nature of the Ca-site in fersmite is quite different to that of microlite or pyrochlore, and crystallographic data confirm that the size of the Ca-site is much smaller in fersmite (average Ca-O bond lengths 2.4 Å [45]) than in pyrochlore (average Ca-O bond lengths 2.58 Å [42]). Furthermore, the Ca-site in pyrochlore is eightfold coordinated [42], but the Ca-site in fersmite is smaller and sixfold coordinated [45]. We find that D_REE_ fersmite/melt are highest in run SE_Pyro2, which was run at the highest final run temperature of 1300 °C, whereas run SE_Pyro6, which was run at 1240 °C, resulted in the lowest D_REE_.

Our data clearly shows that Ce is much more compatible than neighboring REE in runs with the HW starting material, which does not contain F. We can explain the deviant behavior of Ce in these runs, as Ce is a multivalent trace element and under oxidizing conditions Ce does occur as Ce^4+^ and Ce^3+^. Whilst most trivalent REE occupy the A-site (i.e. the Ca-site) in pyrochlores, it could be that the smaller Ce^4+^ ion (ionic radius of 0.87 Å [46]) can also occupy the B-site, which is mainly occupied by pentavalent Nb and Ta. More crystallographic data on pyrochlores would be needed to confirm this notion.

However, the D’s (pyrochlore/melt) in runs with the SE-pyro starting material, which contains F, show no positive Ce-anomaly (Fig. 3), and D (fersmite/melt) with SE-pyro also show only a very weak positive Ce anomaly. We speculate that this behavior may be caused by F in the melt, which may form stable complexes with Ce^4+^, i.e. similar to CeF_8_^4−^ or CeF_6_^2−^ type compounds that have been reported in experiments with aqueous solutions [47]. These complexes in the melt would lead to an increased partitioning of Ce^4+^ into the melt, which consequently lowers D_Ce_ mineral/melt in F-bearing runs (Fig. 3).

**Fig. 4:**
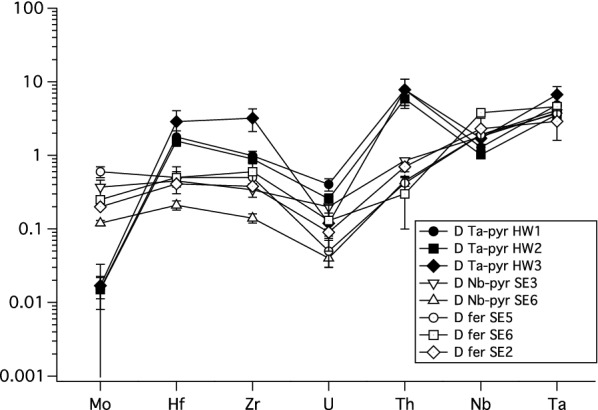
High field strength element partition coefficients between pyrochlore-group minerals, (Ca-Ta-microlite (Ta-pyr), and Ca-Nb pyrochlore (Nb-pyr)), Ca-Nb fersmite (fer), and melts. Minerals and quenched melts were analyzed with LA-ICPMS techniques. See text for details

As to the high field strength elements (HFSE), we find that the HFSE partition coefficients for Nb and Ta are above unity (Table 4), and other D_HFSE_ (Th, Zr, Hf, U, Mo) vary with bulk composition and temperature (Fig. 4). D_Ta_ is very similar to D_Nb_, but overall we find that D_Ta_ are systematically higher than D_Nb_, which is in good agreement with previous data for perovskite, rutile and other Ti–rich oxide minerals [2, 3, 5, 7, 8, 10, 13, 48–50]. Our data shows that D_HFSE_ for Ta-pyrochlore (HW1-3) are mostly above 1, only D_Mo_ in the HW runs are systematically below 1. The Zr and Hf partition coefficients are close to 1, with D_Zr_ and D_Hf_ slightly higher than 1 for Ta-rich microlite, and D_Zr_ and D_Hf_ are slightly lower than 1 for Nb-pyrochlore and fersmite (Table 4).

This compatible in Ta-rich microlite with a partition coefficient of almost 10, c.f., Table 4). Although one might expect U to exhibit similar partitioning behavior as Th, we find that U is slightly incompatible in pyrochlore, microlite and fersmite (Table 4). This can be readily explained by the oxidizing conditions prevailing in our 1-atm experiments, under which U ions occur mainly in the 6 + valence state and, thus, do not fit as well into the mineral structures as U^4+^ would [8, 10]. As Th^4+^ (ionic radius of 0.94 Å) and U^6+^ (with its ionic radius of 0.89 Å in octahedral coordination [46]) are too large to replace Nb or Ta (ionic radius of 0.64 Å [46]) in pyrochlore-group minerals, we presume that both elements partition into the Ca-sites (ionic radius of Ca is 1.0 Å in octahedral coordination), despite the large charge difference.

## Implications

To interpret the trace element budget of alkaline-rich und undersaturated melts, one needs to understand the trace element partitioning of all mineral phases involved. In the next paragraph we will show some important aspects of how crystallizing pyrochlore-group minerals may affect coexisting melt compositions. Note that our experiments were done in silicate melt systems, and as no other data are available, we assume that our results are also applicable to carbonate melt systems.

Our data show that pyrochlores prefer to incorporate Th over U (D_Th_ > D_U_) under oxidizing conditions; hence crystallization of pyrochlore will deplete a melt in Th and enrich it in U. The composition of natural pyrochlores varies dramatically, but many primitive pyrochlores contain superchondritic Th/U (e.g., [51]), which may be caused by the aforementioned uneven partitioning of Th and U. If natural pyrochlores contain a lot more U than Th (e.g. [15]) then either the melt must have had exceptionally high U/Th [52], or, perhaps more common, the pyrochlores were precipitated from or altered by a hydrothermal fluid, which can transport U much more efficiently than Th [15]. A recent paper shows nicely how the U and Th composition of pyrochlore varies with increasing degree of alteration: Whereas primitive pyrochlores contain wt.% of Th and only little U, the U content of pyrochlore increases and the Th content decreases with increasing degree of hydrothermal alteration [51].

Carbonatite and undersaturated silicate rocks are often extremely enriched in REE, so that many carbonatites are mined for the REE. As pyrochlores prefer to incorporate the lighter REE over the heaver ones (D_LREE_ > D_HREE_), precipitation of pyrochlore minerals from a melt will lower the light REE concentrations (i.e., La, Ce, etc.) significantly, whereas the concentrations of the heaver REE (e.g., Yb, Lu) will not be depleted so much (D_HREE_ ≈ 1). This may explain why primary bastnäsite, a Ce-carbonate, is scarce in carbonatites [26], and only secondary hydrothermal alteration (and breakdown of pyrochlores) may lead to the formation of the formation of REE-carbonates.

## Conclusions

We present hitherto unknown experimentally determined trace element partition coefficients between pyrochlore, microlite, fersmite, and silicate melts. The partition coefficients show that pyrochlore, microlite, and fersmite can fractionate U from Th during differentiation of a magma under oxidizing conditions. Furthermore, our REE partition coefficients reveal that pyrochlores prefer to incorporate the lighter REE over the heavy REE, and this may prevent the crystallization of REE-carbonates in many carbonatite melts.

## Data Availability

The dataset supporting the conclusions of this article is included within the article.
